# Characterization of isolated ventricular myocytes from adult zebrafish (*Danio rerio*)

**DOI:** 10.1016/j.bbrc.2008.06.109

**Published:** 2008-09-12

**Authors:** Fabien Brette, Guillermo Luxan, Caroline Cros, Hayley Dixey, Christopher Wilson, Holly A. Shiels

**Affiliations:** Faculty of Life Sciences, University of Manchester, 2nd Floor Core Technology Facility, 46 Grafton Street, Manchester M13 9NT, UK

**Keywords:** Heart, Cardiac myocytes, Ion channels, Zebrafish, Enzymatic isolation

## Abstract

The zebrafish is widely used for human related disease studies. Surprisingly, there is no information about the electrical activity of single myocytes freshly isolated from adult zebrafish ventricle. In this study, we present an enzymatic method to isolate ventricular myocytes from zebrafish heart that yield a large number of calcium tolerant cells. Ventricular myocytes from zebrafish were imaged using light and confocal microscopy. Myocytes were mostly rod shaped and responded by vigorous contraction to field electrical stimulation. Whole cell configuration of the patch clamp technique was used to record electrophysiological characteristics of myocytes. Action potentials present a long duration and a plateau phase and action potential duration decreases when increasing stimulation frequency (as observed in larger mammals). Together these results indicate that zebrafish is a species ideally suited for investigation of ion channels related mutation screening of cardiac alteration important in human.

Mouse is currently the main model used to investigate human diseases. However several aspects of murine biology limit its routine use in large-scale genetic and therapeutic screening. With respect to cardiac physiology, mouse heart is very different from human heart [1]: basal heart rate is 7 to 10 times higher, there is a strong dependence of contraction upon sarcoplasmic reticulum Ca^2+^ release, and different ion channel isoforms are present. The latter leads to a very different electrical activity, characterized by a very short action potential, without a distinct plateau, and no/very little change of action potential duration when increasing pacing rate (see for review [2]). In contrast, in human ventricular myocytes, action potentials are long, with a prominent plateau phase due to the presence of *I*_Kr_ (rapid delayed rectifier), which is absent in mouse. Further, in human and most other large mammals, increasing stimulation frequency decreases action potential duration [3]. Thus, although mouse models of heart disease have provided valuable insight into certain aspects of human cardiac disease, advances in patho-physiological mechanisms of cardiac arrhythmia are hindered by the lack of genetic animal models that exhibit action potential characteristics comparable to human cardiomyocytes; alternative animal models should be looked for. Recently, zebrafish have become the model for many researchers who are interested in embryologically and genetically tractable diseases [4,5]. Indeed, mutagenesis and screening strategies on a large scale are available in this species with an economy that is not possible in other vertebrate systems. Surprisingly, there is no information about cardiac electrical activity of adult ventricular myocytes from zebrafish. In this study, we present a method to isolate adult ventricular myocytes from zebrafish heart and characterize the morphological and electrophysiological properties of these freshly isolated cells.

## Materials and methods

*Isolation of zebrafish ventricular myocytes.* Adult ventricular myocytes were obtained by enzymatic dissociation. All procedures adhere to the guidelines from the United Kingdom Home Office Animals Scientific Procedures Act of 1986 as approved by the Animals Procedures Committee. Zebrafish were stunned by a blow in the head and the brain pithed. The heart was quickly removed and, under a microscope the ventricle was cut from bulbous and atria. Ventricles from 3 fish were pooled together, weighted and placed in a small Petri dish containing 10 ml of the isolation solution: (mM) 100 NaCl, 10 KCl, 1.2 KH_2_PO_4_, 4 MgSO_4_, 50 Taurine, 20 Glucose, and 10 Hepes (pH to 6.9 with NaOH), supplemented with collagenase (type IA, Sigma 0.75 mg/ml), trypsin (type IX-S, Sigma 0.5 mg/ml), and BSA (Sigma, 0.75 mg/ml). Tissue was gently agitated for ∼30 min and iteratively minced with fine scissors. Dissociation was ended by slow centrifugation (via a hand-turned centrifuge) for 1 min and the resulting pellet was re-suspended into ∼3 ml isolation solution. Cells subsequently obtained were stored at room temperature and used within 8 hours. All experiments were performed at room temperature (20–23 °C), i.e., physiological temperature for the zebrafish.

*Confocal imaging.* The cell membrane was visualized by staining with the lipophilic dye di-8-ANNEPS (5 μM for 5 min) and confocal microscopy (Leica SP2 AOBS Confocal Microscope), as previously described [6]. Width measures were taken in at a midpoint of the z-scan. Myocyte depth was calculated from the z-stack images of myocytes. An elliptical cross-sectional area was assumed and volume was calculated as length by cross-sectional area, as described previously [7].

*Cell shortening measurement.* Isolated cells were placed in an experimental chamber on the stage of an inverted microscope (Diaphot, Nikon, Tokyo, Japan). The chamber was continuously perfused with a physiological saline containing (mM): 150 NaCl, 5.4 KCl, 1.5 MgSO_4_, 0.4 NaH_2_PO_4_, 2 CaCl_2_, 10 glucose, 10 HEPES, pH set to 7.7 with NaOH. Cells were field stimulated by external platinum electrodes at a frequency of 0.6 Hz. Video images of the cell were obtained using a camera and Studio 10 Quickstart software (Pinnacle systems, CA) used for movie acquisition on a Pentium PC. Cell length analysis was performed using NIH ImageJ software.

*Electrophysiological recording.* Cells were bathed with a physiological saline containing as described above. Membrane potential and currents were recorded using the whole-cell configuration of the patch clamp technique; settings and properties were as described previously [8]. Briefly, an Axopatch 200B (Axon Instruments, CA) amplifier controlled by a Pentium PC connected via a Digidata 1322A A/D converter (Axon Instruments, CA), was used for data acquisition and analysis using pClamp software (Axon Instruments, CA). Signals were filtered at 2–10 kHz using an 8-pole Bessel low pass filter before digitization at 10–20 kHz and storage. Patch pipette resistance was typically 2–4 MΩ when filled with intracellular solution (in mM: 139 KCl, 10 NaCl, 0.5 MgCl_2_, 5 Mg-ATP, 0.5 EGTA, 10 HEPES, and 0.4 GTPTris, set to pH 7.2 with KOH). Cell membrane capacitance was measured using the “membrane test module” in Clampex (fitting the decay of the capacitance current recorded during a 10 mV depolarizing pulse from a holding potential of −80 mV). Action potentials were evoked by 5 ms sub-threshold current steps. The stimulus frequency was 0.1, 1, and 2 Hz. Action potential duration (APD) was measured as the duration from the overshoot to three different percentages of repolarization (25: APD_25_; 50: APD_50_; 90: APD_90_). Membrane potentials were corrected by −4.5 mV to compensate for liquid junction potentials between the external and pipette solutions.

Na and Ca currents (*I*_Na_ and *I*_Ca_) were measured using external and internal solutions described above. Cell capacitance and series resistance were compensated (>80%) so that the maximum voltage error was <3 mV. Under these conditions, we have previously shown that the voltage control is very good even for large current (i.e. *I*_Na_ ∼15 nA, [9]). *I*_Na_ and *I*_Ca_ were elicited by a double pulse protocol: a rectangular step to −40 mV (100 ms pulse from a holding potential of −80 mV) elicited *I*_Na_ (and inactivate it by the end of the pulse), then a rectangular step to 0 mV (250 ms) elicited *I*_Ca_ Trains of depolarizing pulses were applied at 0.1 Hz. Current–voltage (*I*–*V*) relationship was obtained with a double pulse protocol which consisted of 100 ms duration prepulse from −80 to −40 mV (to activate and inactivate *I*_Na_), and a test pulse from −40 to +60 mV (250 ms; in 10 mV steps). The stimulus frequency was 0.2 Hz. Currents are expressed as current density, i.e., normalized to cell capacitance (pA/pF).

*Chemicals.* All solutions were prepared using ultrapure water supplied by a Milli-Q system (Millipore, Watford, UK). All solution constituents were reagent grade and purchased from Sigma (St. Louis, MO).

*Statistics.* Data are presented as mean ± SE. Statistical analysis was performed using SigmaStat software. Student–Newman–Keuls Method and Friedman Repeated Measures Analysis of Variance on Ranks were used to test the effect of stimulation frequency within the same group of cells. *P* < 0.05 was taken as significant.

## Results and discussion

### Morphological characteristics of zebrafish ventricular myocytes

**Fig. 1:**
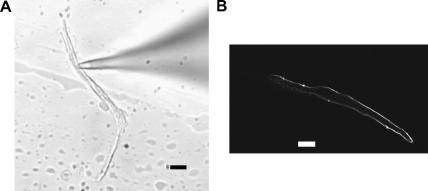
Appearance of adult zebrafish ventricular myocytes under high magnification. (A) Phase contrast image of an isolated myocyte used for patch clamp experiments. (B) Confocal image of a single cardiac myocyte stained with di-8-ANNEPS. Scale bar is 5 μm. All cells were bathed in 2 mM Ca physiological saline solution.

Fig. 1 shows the microscopic appearance of ventricular zebrafish myocytes. Under light microscopy, most cardiac myocytes appeared rod-shaped, as in mammalian myocytes (Fig. 1A). Cross-striations (indicating presence of the sarcomere) are also visible. We next investigated the presence of transverse-tubules (t-tubules) by staining the sarcolemma using a lipophilic dye (di-8-ANNEPS, see Materials and methods) and confocal microscopy. In all ventricular cells studied (*n* = 25), t-tubules were absent (Fig. 1B). This is consistent with a previous report in fish ventricular myocytes (from trout, [7]). Indeed, t-tubules appear to be present in ventricular myocytes from mammals only (see for review [10]). We used these confocal images to measure ventricular myocytes length, width and depth (see Materials and methods). Ventricular myocytes were 100.4 ± 3.7 μm long, 4.6 ± 0.3 μm wide and 6.0 ± 0.3 μm depth (*n* = 25). From these values, we calculated a volume of 2.2 ± 0.2 pL. These morphological characteristics are similar to investigation from ventricular myocytes in other fish species studied so far [11].

### Functional investigation of zebrafish ventricular myocytes

To test the viability of the isolated myocytes, we investigated the response of these cells to field electrical stimulation. Cells contracted vigorously at each stimulation (see movie on the online data supplement), indicating that cells are healthy. On average, cell shortening was 6.0 ± 1.3% of resting cell length (*n* = 7) at 0.6 Hz.

### Electrophysiological characteristics of adult ventricular zebrafish myocytes

Electrophysiological properties of the isolated myocytes were studied using the patch clamp technique. Cell capacitance was 26.0 ± 1.1 pF (*n* = 69). This value is similar to previous investigations in ventricular myocytes from other fish species (e.g. [12]). When cell capacitance value is combined with cell volume (above), the surface to volume ratio is ∼12.

**Fig. 2:**
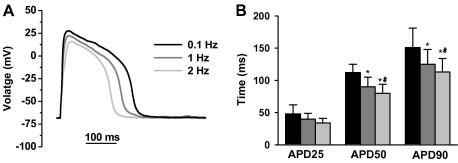
Action potential characteristics of isolated ventricular myocytes from zebrafish. (A) Representative action potentials recorded at 0.1 Hz (black trace), 1 Hz (gray trace), and 2 Hz (light gray trace) in the same cell. (B) Mean (±SEM) action potential duration at 25%, 50%, and 90% repolarisation (APD_25_, APD_50_, and APD_90_) at 0.1 Hz (black bars), 1 Hz (gray bars), and 2 Hz (light gray bars). Data are from 14 myocytes. ^∗^ Indicates *P* < 0.05 versus 0.1 Hz and ^#^ indicates *P* < 0.05 versus 1 Hz.

Fig. 2A shows representative action potential recorded at 0.1 (black), 1 (gray), and 2 (light gray) Hz in an isolated ventricular myocyte from zebrafish. At 0.1 Hz stimulation frequency, resting membrane potential was −70.4 ± 2.8 mV and APD_25_, APD_50_, and APD_90_ was 48 ± 14, 112 ± 23, 151 ± 30 ms, respectively (*n* = 14). The configuration of the action potential closely resemble those from large mammals ventricular myocytes, notably human (e.g., see fig. 1 from [13]), with the clear presence of a plateau phase. The zebrafish action potentials strongly contrast to the triangular action potential observed in small rodents (i.e., rat and mouse, see for review [3]). Increasing stimulation rate to 1 and 2 Hz significantly decrease APD_50_, and APD_90_ (Fig. 2B, *n* = 14, *P* < 0.05). This feature is characteristics of large mammals [2].

**Fig. 3:**
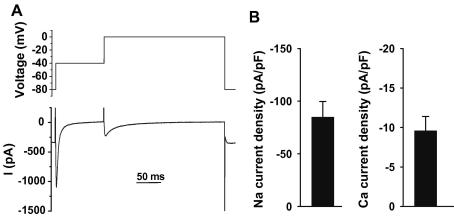
Na and Ca currents in isolated ventricular myocytes from zebrafish. (A) Representative membrane current recorded at −40 mV (Na current) and 0 mV (Ca current). The inset shows the voltage protocol. (B) Mean (±SEM) Na current density (left) and Ca current density. Data are from 12 myocytes.

**Fig. 4:**
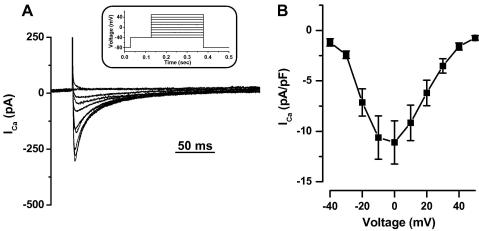
Ca current–voltage relationship in isolated ventricular myocytes from zebrafish. (A) Representative Ca currents recorded from −40 to +50 mV in 10 mV steps. The inset shows the voltage protocol. (B) Mean (±SEM) Ca current–voltage relationship. Data are from 10 myocytes.

We next investigated the characteristics of *I*_Na_ and *I*_Ca_ using a double pulse protocol (see Materials and methods and Fig. 3A). Typical results are shown in the lower panel of Fig. 3A. Mean data (*n* = 12) are presented on fig. 3B and indicate that *I*_Na_ density (−84.8 ± 14.9 pA/pF) is ∼4 times smaller than in cardiac myocytes from mammals [3]; in contrast, *I*_Ca_ density (−9.58 ± 1.81 pA/pF) is similar to other cardiac species (fish [11] and mammals [14]). To investigate this further, we recorded *I*–*V* relationship for *I*_Ca_. Fig. 4A shows representative *I*_Ca_ recorded from −40 to +50 mV (voltage protocol in the inset). Mean data indicates that *I*_Ca_–*V* curves are bell-shaped and voltage characteristics similar to other cardiac species (fish [11] and mammals [14]).

### Impact of the study

Our study provides an enzymatic method to obtain calcium tolerant isolated ventricular myocytes from the adult zebrafish heart. We show that these myocytes respond to field stimulation by contracting vigorously and can be used for electrophysiological experiments (patch clamp, Fig. 2–4). A previous study has described a method to isolate myocytes from adult zebrafish [15]. However, in this study, investigators recorded only few electrophysiological properties (pacemaker current and *I*_Na_) of myocytes kept in culture. Clearly, this can complicate the interpretation of the data because of the morphological and genomic changes which occur during primary culture in mammalian myocytes (e.g. [16]) and fish myocytes [17]. A very recent study has used zebrafish to investigate cardiac related diseases, e.g., long QT syndrome [18]. In this study, electrophysiological properties of single embryonic myocytes were recorded, and it can be difficult to extrapolate to adult because of the physiological differences between embryonic and adult myocytes (see for review [3]). Thus, our method enables other investigators to explore electrophysiological properties of *freshly* isolated *adult* myocytes, minimizing morphological and genomic changes. Importantly, our data shows that action potential characteristics from myocytes of adult zebrafish closely resemble those of human myocytes (Fig. 2). This suggests that zebrafish provide an experimental system to explore the molecular mechanisms underlying patho-physiological changes in heart function, and complement mouse studies which are limited by their action potential characteristics (see Introduction). To conclude, we believe that the method described in this paper will allow further investigation of ion channel related mutation screening in an emerging model organism of cardiac alterations important in human cardiac physiology.
